# Probing the interaction of lysozyme with cardiac glycoside digitoxin: experimental and *in silico* analyses

**DOI:** 10.3389/fmolb.2023.1327740

**Published:** 2023-12-22

**Authors:** Mohd Sajid Ali, Hamad A. Al-Lohedan, Rittik Bhati, Jayaraman Muthukumaran

**Affiliations:** ^1^ Surfactant Research Chair, Department of Chemistry, College of Science, King Saud University, Riyadh, Saudi Arabia; ^2^ Department of Biotechnology, Sharda School of Engineering and Technology, Sharda University, Greater Noida, India

**Keywords:** lysozyme, digitoxin, fluorescence, molecular docking, molecular dynamics simulations

## Abstract

Digitoxin is a cardiac glycoside used to treat heart failure and heart arrhythmia. However, its therapeutic concentration range is very narrow. High doses of digitoxin are associated with severe side effects; therefore, it is necessary to develop the delivery system which can control the plasma levels of it. In this context, the binding of lysozyme, an important protein having many applications, with digitoxin has been studied to see the ability of the former as a carrier. The studies were carried out using both experimental and computational methods. The intrinsic fluorescence of lysozyme increased on the addition of digitoxin. Fluorescence results suggested that there was a strong interaction between lysozyme and digitoxin which was favored, mainly, by hydrophobic forces. Further, digitoxin affected the secondary structure of lysozyme slightly by causing the partial unfolding of lysozyme. The preferred binding site of digitoxin within lysozyme was the large cavity of the protein. Molecular docking studies also established the principal role of hydrophobic forces in the binding with a significant support of hydrogen bonding. Frontier molecular orbitals of free digitoxin and in complexation with lysozyme were also computed and discussed. The findings from molecular dynamics simulation studies elucidate that, when contrasted with the first and third conformations of the digitoxin-bound lysozyme complex, the second conformation promotes structural stability, reduces flexibility, and enhances the compactness and folding properties of lysozyme. The overall study shows that lysozyme could act as a potential carrier for digitoxin in pharmaceutical formulations.

## 1 Introduction

Interactions of proteins with drugs are important in many ways and have diverse applications. Being a receptor to the drug is one of the most common characteristics for many proteins (Attie and Raines, 1995; Li et al., 2023). Moreover, proteins can act as potential carriers of the drug and that’s why these are very common substances for the drug-delivery formulation owing to their varieties and biocompatibility, in general. Proteins have several binding sites within themselves that engage them to bind various substances with different affinities (Konc and Janežič, 2022). The complex structures of the proteins are also sensitive to the environment which is another feature for their efficient role as drug-carrier agents (Macdonald and Johnson, 2001; Yin et al., 2023). Another important aspect of this field is how a drug is affecting the structure of the protein. A drug consumed by mankind goes inside the human body to perform its therapeutic action where it binds with numerous proteins (Lalatsa et al., 2011). Understanding the binding mechanism of a drug and protein and the effect of the former on the latter’s structure and function is also an important topic which must also be considered.

Lysozyme is an important globular protein known to have tremendous biological and pharmaceutical applications (Swaminathan et al., 2011; Ferraboschi et al., 2021). t is an enzyme, also known as muramidase, which is responsible for killing Gram positive bacteria by breaking their cell wall. Lysozyme has many natural occurrences which include mucus, tears, and human milk. It is also found in human blood in small quantities (Hankiewicz and Swierczek, 1974). The use of lysozyme in drug delivery formulations has also been reported. Lysozyme is a single polypeptide protein containing 129 amino acids and having around 14.3 kDa molecular weight.

**SCHEME 1:**
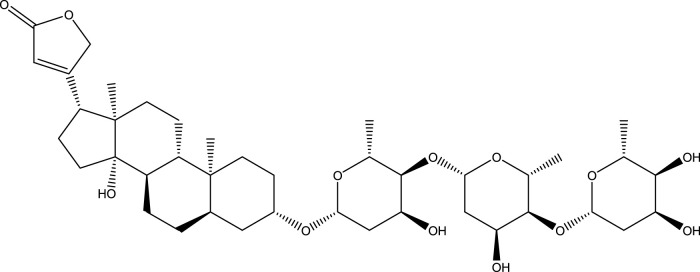
Chemical structure of digitoxin.

Digitoxin (Scheme 1) is a steroidal cardiac glycoside, obtained from the plant *Digitalis purpurea*, used for the treatment of heart failure and certain kinds of heart arrhythmia. It is usually prescribed to patients with chronic cardiac insufficiency, particularly those with impaired renal function (Bavendiek et al., 2019). Studies have shown that digitoxin can be a potential candidate for the treatment of several types of cancers (Hosseini et al., 2019; Pollard et al., 2019; Eldawud et al., 2020; Gan et al., 2020; Chou et al., 2021). Furthermore, digitoxin has also shown a probable candidate for the treatment of COVID-19 by blocking a virally-activated cytokine storm, an immune response which resulted in the multiorgan injury, starting with the lungs, that leads to critical illness and death (Pollard et al., 2020). The digitoxin therapy has limitations due to its narrow plasma concentration range of 10–30 ng/mL (Cha et al., 2020). A higher concentration of digitoxin may lead to the serious toxicity which can cause serious emergencies like severe vomiting, nausea, and malaise (Iraci et al., 2023). Therefore, it is necessary to develop the suitable delivery agent for the delivery of digitoxin which can control its plasma level within the benign range. To understand whether lysozyme can be used in the drug-delivery formulations containing digitoxin, we have designed the present work and studied their interaction using experimental and computational methods.

## 2 Materials and methods

Lysozyme (≥98%, L4919) and digitoxin were the products of Sigma, United States of America. The stock solutions of proteins were prepared in the 20 mM tris buffer of pH 7.4 which was also the medium of all the studies. The stock solution (5 µM) of digitoxin was made in absolute ethanol to minimize the concentration of solvent (1%). The effect of solvent was also seen on the pure lysozyme and it was found that 1% ethanol did not influence the UV-visible, far UV CD and fluorescence spectra of lysozyme. The studies were carried out at 25°C temperature unless stated otherwise. UV-visible studies were performed on Perkin Elmer Lambda 45 spectrophotometer within the range of 200 nm–500 nm using the quartz cuvettes. For every measurement, the concentration of digitoxin was identical in blank as well as in sample cell to get the actual absorbance from the lysozyme. Intrinsic fluorescence measurements were performed on Hitachi F 7000 spectrofluorometer equipped with the programmable temperature controller using a transparent quartz cell. The protein solution was excited at 295 nm and the emission fluorescence intensity was recorded from 300 nm to 500 nm. The Rayleigh light scattering experiments were performed by exciting the protein at 350 nm and the emission was also recorded at the same wavelength using the synchronous fluorescence setting of the instrument. The excitation and emission slit widths were adjusted to 5 nm with a PMT voltage of 500 V. The circular dichroism studies of lysozyme in presence of digitoxin were carried out with JASCO J-815 spectropolarimeter equipped with a Peltier-type temperature controller. The instrument was calibrated with d-10-camphorsulfonic acid. All the CD spectra were collected in a cell of 1 mm path-length. The scan speed was 100 nm/min and response time of 1 s for all measurements. Each spectrum was the average of 3 scans. AutoDock Vina 1.1.2 (Molecular Graphics Lab, La Jolla, United States of America) was used to see the possible binding mode of digitoxin with lysozyme (Trott and Olson, 2010). The 3D structure of lysozyme (PDB ID: 2LYZ) was obtained from the Protein Data Bank (PDB). The 3D structure of digitoxin was obtained from Pubchem database. The AutoDockTools 1.5.6 package (Molecular Graphics Lab, La Jolla, United States of America) was used to generate the docking input files (Morris et al., 2009). The search grid for blind docking of the lysozyme was identified as center_x = −0.49, center_y = 20.577, center_z = 19.266 with dimensions size_x = 40, size_y = 40, size_z = 40. The exhaustiveness in this case was 1000 which allows drug molecules to move almost at every possible site at lysozyme. The least energy conformation was selected among 20 conformations obtained through docking. The geometry of digitoxin was optimized using the ORCA program. For free digitoxin, the structures which have been described in docking section were used and complexed digitoxin was obtained from the digitoxin-lysozyme complex obtained through molecular docking. Discovery studio visualizer was used to remove lysozyme molecule from the complex. The orca input file was generated using Avogadro platform, in the first step, auto optimization was performed using universal force field using four steps per update and steepest descent algorithm. Following that orca input file was generated using advanced option in orca input parameters setting tab. Geometry optimization was performed using DFT method with B3LYP functional.

Apart from the molecular docking and DFT calculation, we have performed performed Molecular Dynamics (MD) simulations on unbound and three digitoxin-bound Lysozyme complexes (first, second and third conformations of digitoxin bound lysozyme complexes) using GROMACS Version 2021.3 (Van Der Spoel et al., 2005). Utilizing in-built commands of Gromacssuch as *gmx pdb2gmx*, *gmx editconf*, and g*mx solvate,* GROMACS-formatted files (GRO, TOP and POSRE) were generated, cubic boxes created, and solvation achieved with four point Tip4p water model. Counter ions (positive or negatively charged) for charge neutrality were added using *gmx genion* command. The topology file for digitoxin was generated by SwissParamweb server (Bugnon et al., 2023). Energy minimization and NVT, NPT equilibration (3 ns) steps were executed using *gmx grompp* and *gmx mdrun* commands. Subsequently, 100 ns production MD simulations were conducted, and structural analyses were performed using various GROMACS commands on corrected trajectories of unbound and three digitoxin bound lysozyme complexes, considering following structural parameters, namely, Root Mean Square Deviation (RMSD), Root Mean Square Fluctuation (RMSF), Radius of Gyration (Rg), Solvent-Accessible Surface Area (SASA), intermolecular hydrogen bonds, and secondary structural analysis. In addition to Global dynamics, we have additionally performed essential dynamics based on Prinicipal Component Analysis of unbound and three digitoxin bound lyozyme complexes.

## 3 Results and discussions

### 3.1 UV-visible absorption spectroscopy

**FIGURE 1:**
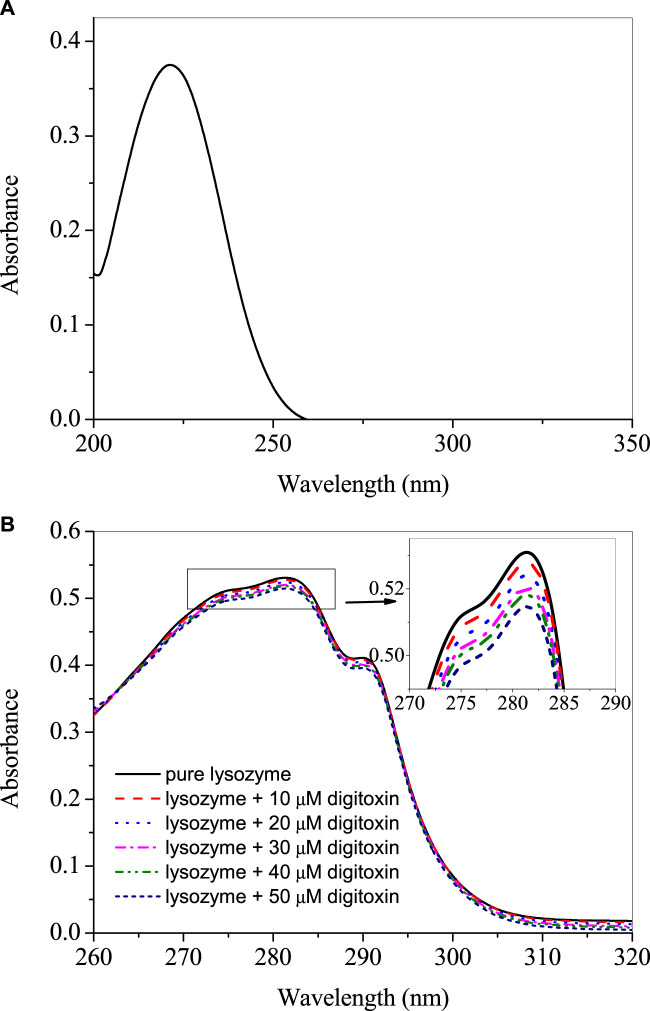
**(A)** UV-visible spectra of 25 µM digitoxin at 25°C. **(B)** Difference UV-visible spectra of lysozyme in presence of various concentrations of digitoxin.

The UV-visible spectrum of digitoxin is given in Figure 1A which shows that it has a strong absorption peak at 225 nm. The UV-visible absorption profiles of lysozyme with and without digitoxin are displayed in Figure 1B. UV-visible spectroscopy can give a preliminary idea about the complex formation between a biomacromolecule and a small molecule or ligand (Ali et al., 2021; Sajid Ali and Al-Lohedan, 2023). Lysozyme, like most of the proteins, has aromatic amino acids (tryptophan, tyrosine and phenylalanine) present in its protein backbone. These amino acids show a strong absorption at 280 nm and any change in the microenvironment of these amino acids could be monitored by scrutinizing the changes in the UV-visible profile of the protein. As can be seen from Figure 1B that there is a slight decrease in the absorption (which is more clearly shown in the inset of the figure), it gives an idea about the complex formation between lysozyme and digitoxin.

### 3.2 Intrinsic fluorescence

**FIGURE 2:**
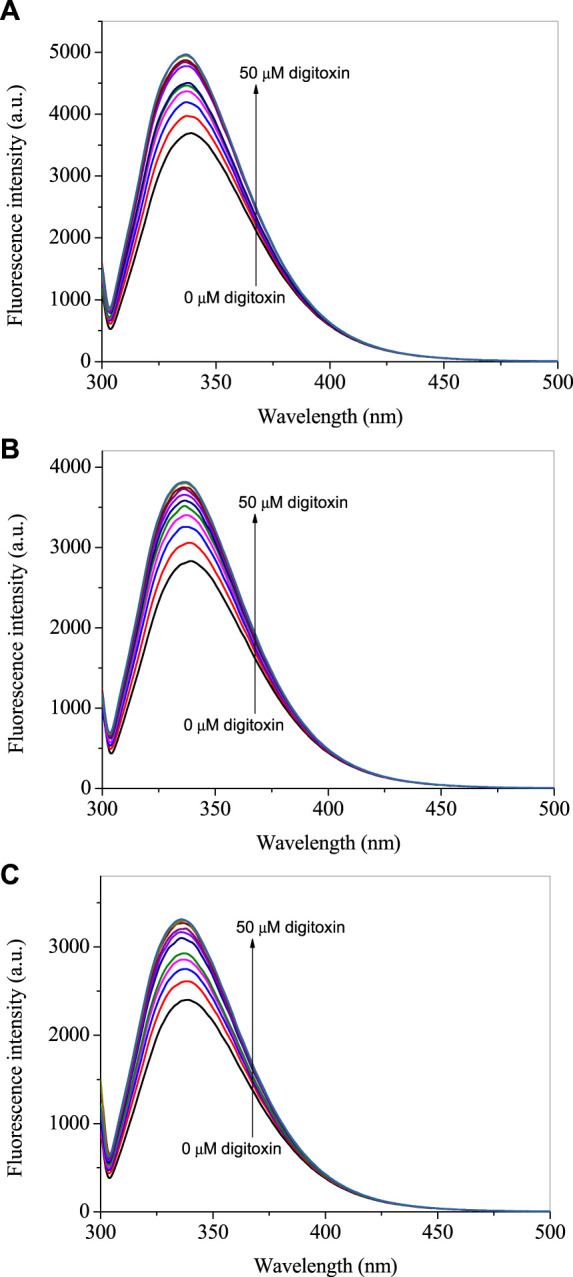
Fluorescence emission spectra of lysozyme in presence of several concentrations of digitoxin ranging from 0 to 50 µM with a constant increment of 5 μM at the excitation wavelength of 295 nm at 25 **(A)**, 35 **(B)** and 45°C **(C)**.

The intrinsic fluorescence of lysozyme is contributed, mainly, by tryptophan, although tyrosine and phenylalanine are also fluorescent amino acids but these have very small and negligible quantum yield, respectively, as compared to the former (Lakowicz, 2006). Therefore, tryptophan fluorescence could be utilized to see changes in the microenvironment of the fluorophore due to the interaction of proteins with the ligands. The fluorescence emission spectra of lysozyme in absence and presence of digitoxin at 295 nm excitation wavelength are given in Figures 2A–C at 25, 35°C and 45°C. Since digitoxin has no absorbance at 295 nm (Figure 1A), the chances of inner filter effect due to its absorption were zero. The fluorescence intensity of lysozyme increased gradually on increasing the concentration of digitoxin which is an indication of the interaction between them as digitoxin did not show any fluorescence emission under these conditions, thus, the fluorescence emission is solely from the contribution of the tryptophan residues present in the lysozyme. This type of fluorescence enhancement has also been seen in our recent studies (Ali and Al-Lohedan, 2023; Sajid Ali and Al-Lohedan, 2023), further, Zhao et al. have also reported that the intensity of bovine serum albumin was also increased in presence of titanate nanotubes (Zhao et al., 2014).

### 3.3 Analysis of fluorescence data

**FIGURE 3:**
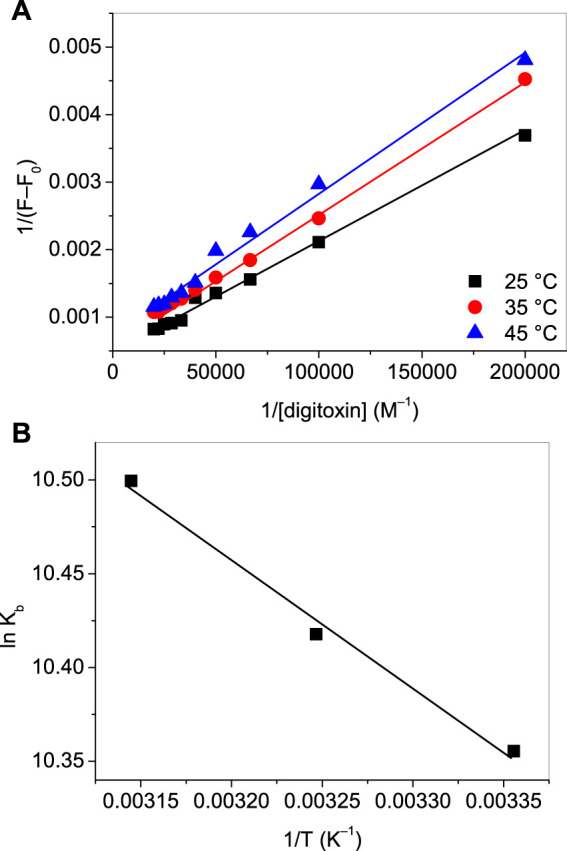
**(A)** Plots of 1/(F–F_0_) vs. 1/[digitoxin]. **(B)** Van ‘t Hoff plots for the interaction of lysozyme with digitoxin.

The binding constant (K_b_) was evaluated using the values of fluorescence intensity change (ΔF = F–F_0_)with respect to the change in the concentration of digitoxin using following equation (Bhattacharyya et al., 1994; He et al., 2006; Valdebenito et al., 2015):

$$\frac{1}{∆F}=\frac{1}{{∆F}_{\max}}+\frac{1}{K_{b}\left[digitoxin\right]}\times \frac{1}{{∆F}_{\max}}$$ (1)

where ΔF_max_ is the maximum change in the fluorescence; F_0_ and F are the fluorescence intensities of lysozyme at 340 nm in absence and presence of digitoxin. The plots of 1/(F-F_0_) vs. 1/[digitoxin] (Figure 3A) could be used to evaluate the K_b_. The calculated values of binding constants of lysozyme-digitoxin interaction are given in Table 1 which show that the interaction between them was a strong one which increases on increasing the temperature.

**TABLE 1:** Analyzed values of binding parameters and thermodynamic parameters for the interaction of lysozyme with digitoxin at various temperatures.

Temperature (°C)	Binding parameters		Thermodynamic parameters		
	K_b_ (M^–1^)	*R* ^2^	∆*G* ^0^ (kJ mol^-1^)	∆*H* ^0^ (kJ mol^-1^)	*∆S* ^0^ (J mol^-1^ K^−1^)
25	3.1×10^4^	0.9950	−25.6	5.6	104.8
35	3.3×10^4^	0.9979	−26.6		
45	3.6×10^4^	0.9915	−27.7		

### 3.4 Estimation of thermodynamic parameters

Studying a process or interaction at various temperatures could be used to evaluate various thermodynamic parameters like free energy change (ΔG), enthalpy change (ΔH) and entropy change (ΔS) using Van ‘t Hoff equations given as:

$$\ln K_{b}=\frac{-\Delta H}{RT}+\frac{\Delta S}{R}$$ (2)

ΔG=ΔH−TΔS (3)

where R is gas constant and T is temperature in K.

These thermodynamic parameters can explicate the feasibility of the interaction as well as give the idea about the types of forces accompanying that process or interaction. A process is considered spontaneous if the values of ΔG are negative. ΔH and ΔS comprise the information regarding the types of forces involved depending on their positive and negative values. When the values of these parameters are positive, the interaction is known to be reinforced by hydrophobic forces, while hydrogen bonding and Van der Waals forces dominate if the values of these parameters are negative (Ross and Subramanian, 1981). The plot of ln K_b_ vs. 1/T (Van ‘t Hoff plot) are given in Figure 3B and the values of thermodynamic parameters are displayed in Table 1. The obtained values of thermodynamic parameters ascertain that the binding between lysozyme and digitoxin is a spontaneous one which is supported by the dominance of hydrophobic forces.

### 3.5 Far-UV circular dichroism (CD) spectroscopy

**FIGURE 4:**
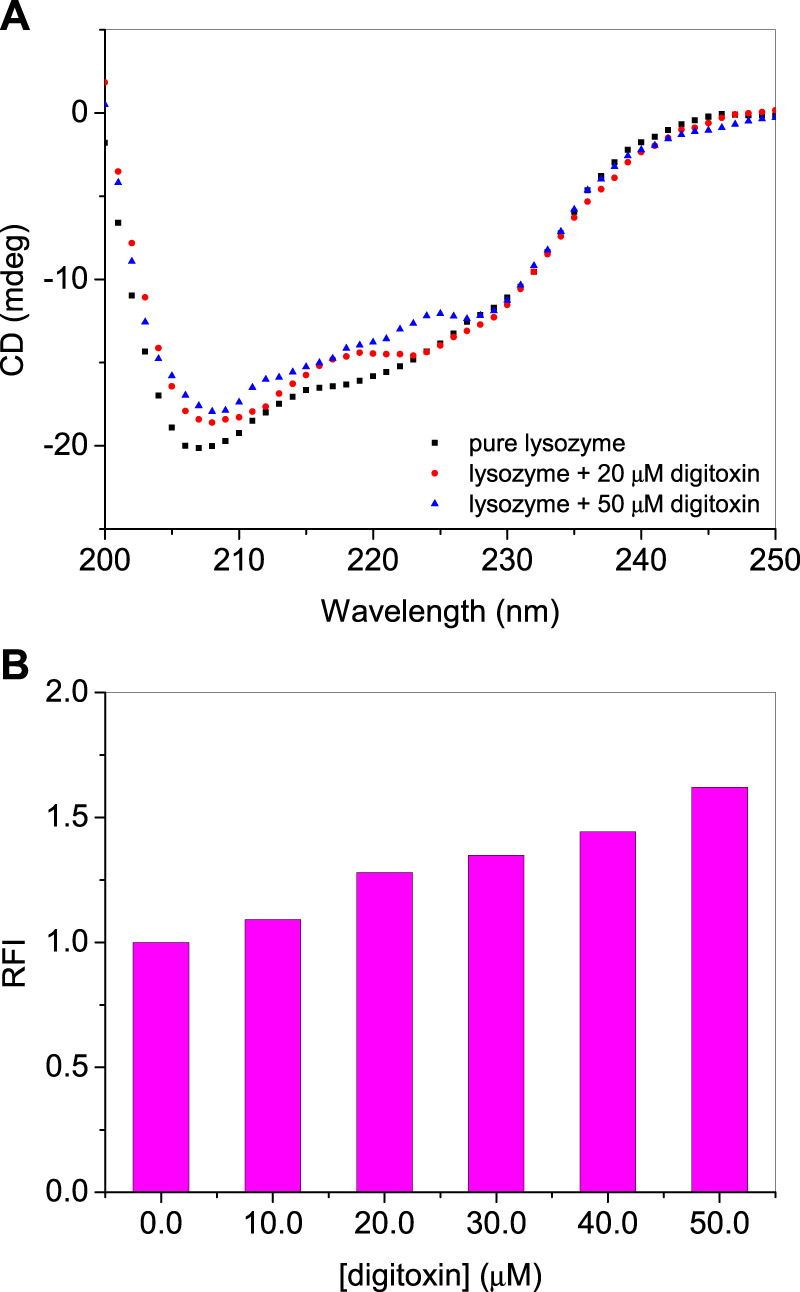
**(A)** Far-UV CD spectra of lysozyme in absence and presence of digitoxin at 25°C. **(B)** RLS intensities of lysozyme-digitoxin system.

Secondary structure of a protein is an important feature which determines their properties and functions. Interacting ligands have interesting effects on the secondary structures of the proteins. A ligand may stabilize or unfold a protein and sometimes there is no significant effect of the ligand on the secondary structure. Far-UV CD spectroscopy is an important technique to determine the secondary structure of the protein. It is reported that lysozyme contains around 40% α-helices (Anderle and Mendelsohn, 1987; Cai and Singh, 1999). The far-UV CD spectra of lysozyme with or without digitoxin are given in Figure 4A. It is evident from the figure that on the addition of digitoxin the α-helicity of lysozyme decreased slightly which is due to the partial unfolding of the protein. The effect of digitoxin on the overall size of the protein was also determined using Rayleigh light scattering (RLS) given in the next section.

### 3.6 Rayleigh light scattering

As described in the CD section that lysozyme undergoes partial unfolding in presence of digitoxin, RLS studies have also performed to know the qualitative effect of the drug on the size of the protein (Ma et al., 1996; Ma et al., 1997). The relative sizes of the protein can be determined using the RLS method in which the protein is excited and emitted at the same wavelength which is 350 nm (Ali et al., 2015; Ali and Al-Lohedan, 2016). The scattering intensity increases as the size of the protein increases on unfolding. Larger the size of the protein or its aggregates greater will be the scattering intensity and *vice versa*. If the change in the intensity is not so large it can be synchronized with the partial unfolding (Ali and Al-Lohedan, 2020). The relative fluorescence intensities (RFIs) of RLS of lysozyme with or without digitoxin are given in Figure 4B. A slight increase in the scattering intensity is due to the slight increase of the size of the protein that matches with the observations obtained from CD studies.

### 3.7 Molecular docking

**FIGURE 5:**
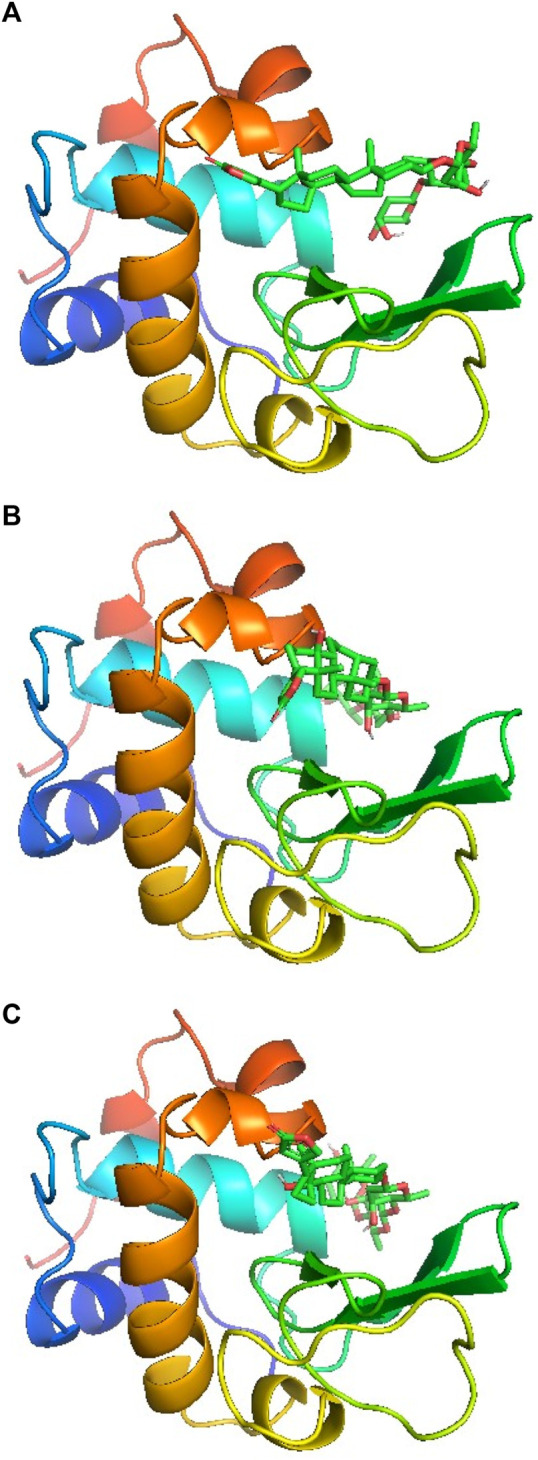
Docking poses of lysozyme with digitoxin: conformer 1 **(A)**, conformer 2 **(B)** and conformer 3 **(C)**.

Molecular docking is an easy and important computational tool to understand the binding site of a ligand within a protein or receptor. In this method, the protein is kept fixed while ligand is allowed to move around the molecule to fit into its most suitable binding site which is then generated according to the least estimated binding free energy values. Since a molecule has several degrees of freedom and flexibilities, a number of conformers are generated depending on the overall binding free energies. The molecular docking simulations for lysozyme-digitoxin system have also been performed and the best three conformers according to their estimated binding free energies have been selected for the discussion. The binding poses are displayed in Figures 5A–C for the conformer 1, 2 and 3, respectively, which show that the most preferred binding site of digitoxin within lysozyme is the big hydrophobic cavity which is also the binding site for most of the molecules (Panja and Halder, 2016; Ali and Al-Lohedan, 2020; Ali et al., 2021; Ali and Al-Lohedan, 2023; Sajid Ali and Al-Lohedan, 2023).

**FIGURE 6:**
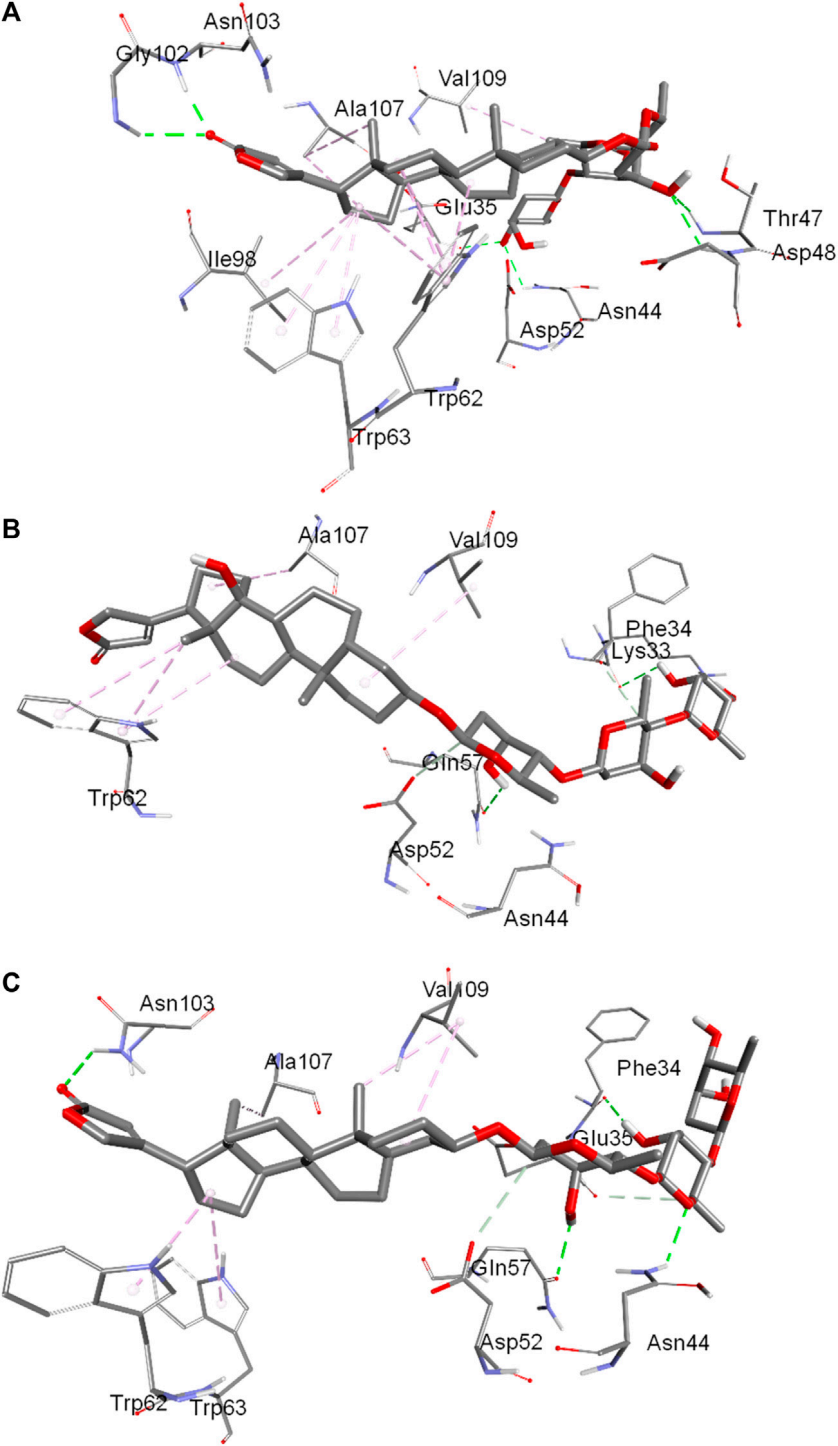
Binding pocket of digitoxin inside lysozyme showing interacting amino acids:conformer 1 **(A)**, conformer 2 **(B)** and conformer 3 **(C)**.

The binding pockets showing interacting amino acids have also been shown in Figures 6A–C for the conformer 1, 2 and 3, respectively. In conformer 1 there were ten hydrophobic interactions in total and six hydrogen bonds. TRP62 was the biggest contributor with four hydrophobic interactions followed by TRP63 and ALA107 each of which have two hydrophobic bonds. The other amino acids involved in hydrophobic forces were VAL109 and ELE98. The hydrogen bonding interaction in conformer 1 was facilitated by ASN44, THR47, ASP48, GLY102, ASN103 and GLU35. In conformer 2, a total of five hydrophobic interactions were reported in which three were contributed by TRP62 while other two were from ALA107 and VAL109. GLN57 and LYS33 were the residues which interacted through hydrogen bonding in conformer 2. In the conformer 3, there were a total of five hydrophobic interactions in which two were contributed by VAL109 while TRP62, TRP63 and ALA107 bonded through one hydrophobic interaction. Hydrogen bonding in this case was facilitated by PHE34, ASN44, GLN57 and ASN103. From the collective information obtained through all three conformers about the involvement of residues it can be deduced that residues like TRP62, TRP63 and VAL109 played important role in the binding through hydrophobic interactions while residues like ASN44, GLN57 and ASN103 were the favored amino acids responsible for hydrogen bonding interactions. Further, hydrophobic interactions were greater in numbers in comparison to the hydrogen bonding interactions that support the experimental findings.

### 3.8 Frontier molecular orbitals (FMOs)

**FIGURE 7:**
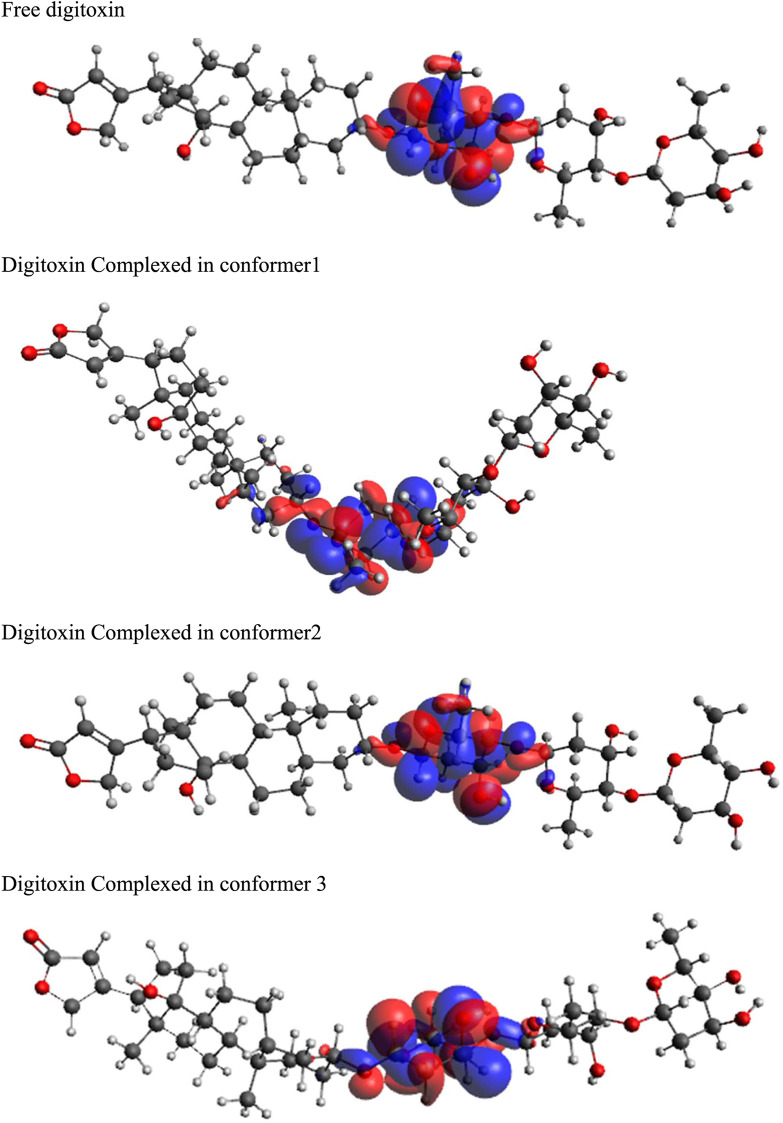
HOMO diagrams of free and complexed digitoxin.

**FIGURE 8:**
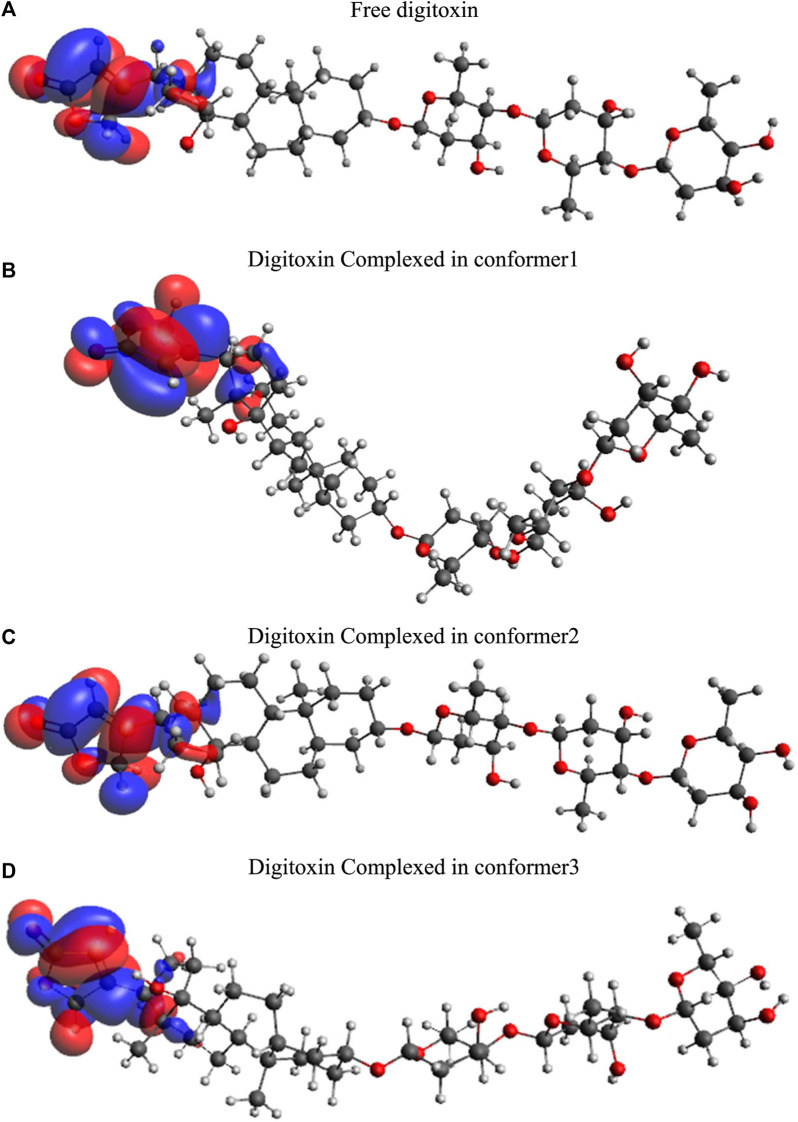
LUMO diagrams of free and complexed digitoxin.

Highest occupied molecular orbitals (HOMO) and lowest unoccupied molecular orbitals (LUMO) have been evaluated for the digitoxin in free form and complexed in the conformers described in the preceding section. The HOMO diagrams of the digitoxin are given in Figure 7. It is discernible that the HOMOs are distributed at the center of the digitoxin with some change in the location and sizes of the orbitals in every form of digitoxin. Although, the spatial arrangements of the atoms and bonds have changed significantly. In contrast, LUMOs (Figure 8) in every form of digitoxin were located at only one terminal position of digitoxin near the furanone ring and its adjacent atoms.

The energy gap between HOMO and LUMO was obtained using following equation:

$$\Delta E=E_{LUMO}-E_{HOMO}$$ (4)

The chemical potential (*µ*) and chemical hardness (*η*) of the system can be calculated as:

$$\mu =\frac{E_{LUMO}+E_{HOMO}}{2}$$ (5)

$$\eta =\frac{E_{LUMO}-E_{HOMO}}{2}$$ (6)

Generally, theionization potential (*I*) is defined as the −*E*_*HOMO*_ value, while the electron affinity (*A*) is equal to −*E*_*LUMO*_. Thus, the electronegativity (*χ*) and electrophilicity (*ω*) can be calculated according to the following equations:

$$\chi =\frac{I+A}{2}$$ (7)

$$\omega =\frac{\mu^{2}}{2\eta }$$ (8)

The parameters calculated using the above equations are given in Table 2.

**TABLE 2:** Frontier molecular orbitals and corresponding parameters obtained through DFT calculations.

	Free digitoxin	Digitoxin in conformer 1	Digitoxin in conformer 2	Digitoxin in conformer 3
HOMO	−6.507	−6.41	−6.541	−6.509
LUMO	−0.933	−0.911	−0.85	−1.36
Energy gap (∆E)	5.574	5.499	5.691	5.149
Chemical potential (µ)	−3.72	−3.6605	−3.6955	−3.9345
Global hardness (η)	2.787	2.7495	2.8455	2.5745
Ionization potential (I = -E_HOMO_)	6.507	6.41	6.541	6.509
Electron affinity (A = -E_LUMO_)	0.933	0.911	0.85	1.36
Electronegativity ((*χ* = (I + A)/2))	3.72	3.6605	3.6955	3.9345
Electrophilicity ((*ω* = μ^2^/2η))	2.482669537	2.436672168	2.399704841	3.006465382

### 3.9 Molecular dynamics simulations

**FIGURE 9:**
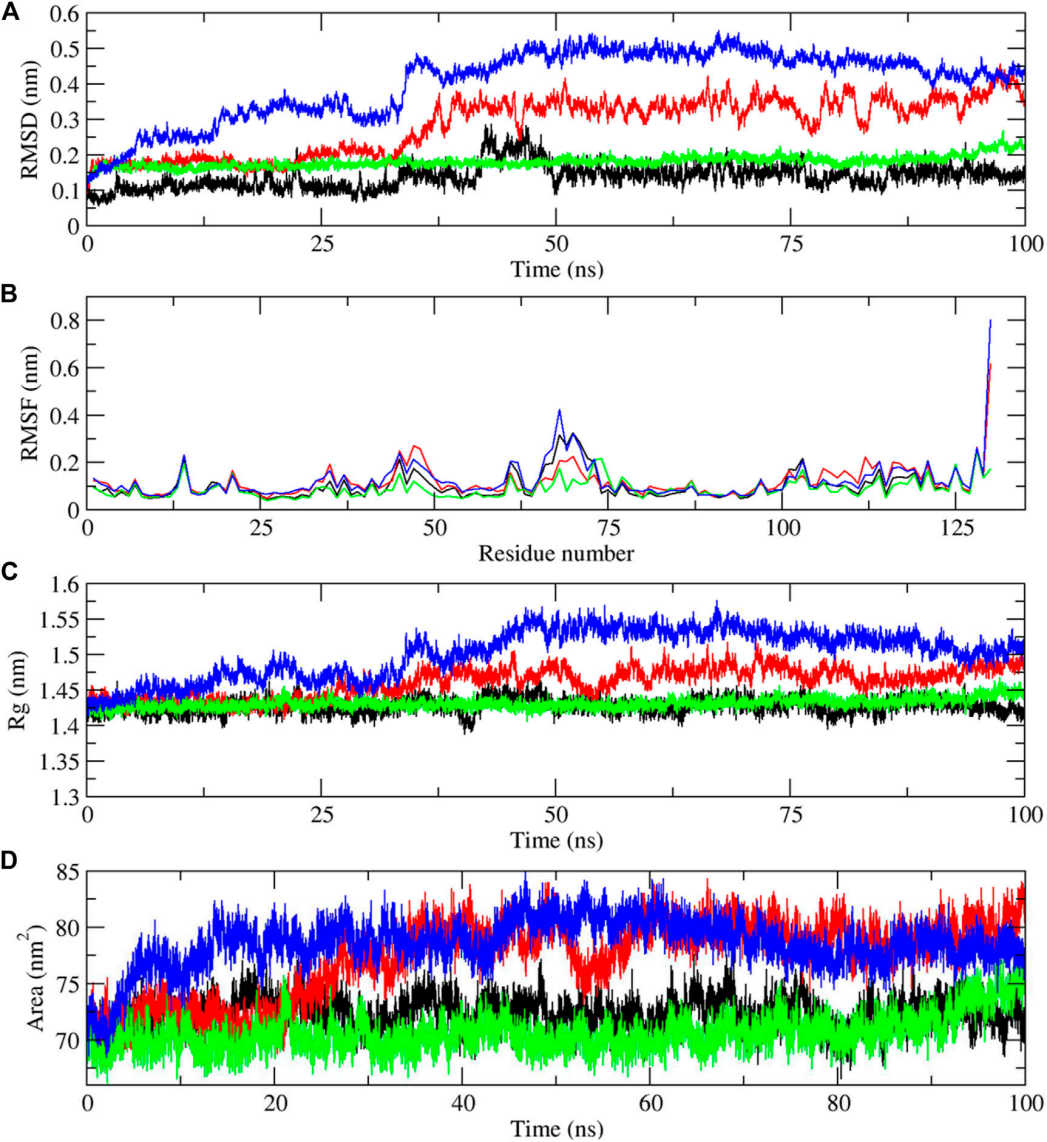
Molecular dynamics simulation results of unbound and first, second and third conformation of digitoxin bound lysozyme complexes, **(A)** Root Mean Square Deviation (RMSD), **(B)** Root Mean Square Fluctuation (RMSF), **(C)** Radius of Gyration (Rg) and **(D)** Solvent-Accessible Surface Area (SASA) (Color codes: Lysozyme-black, first conformation of digitoxin bound lysozyme complex-red, second conformation of digitoxin bound lysozyme complex-green and third conformation of digitoxin bound lysozyme complex-blue).

**FIGURE 10:**
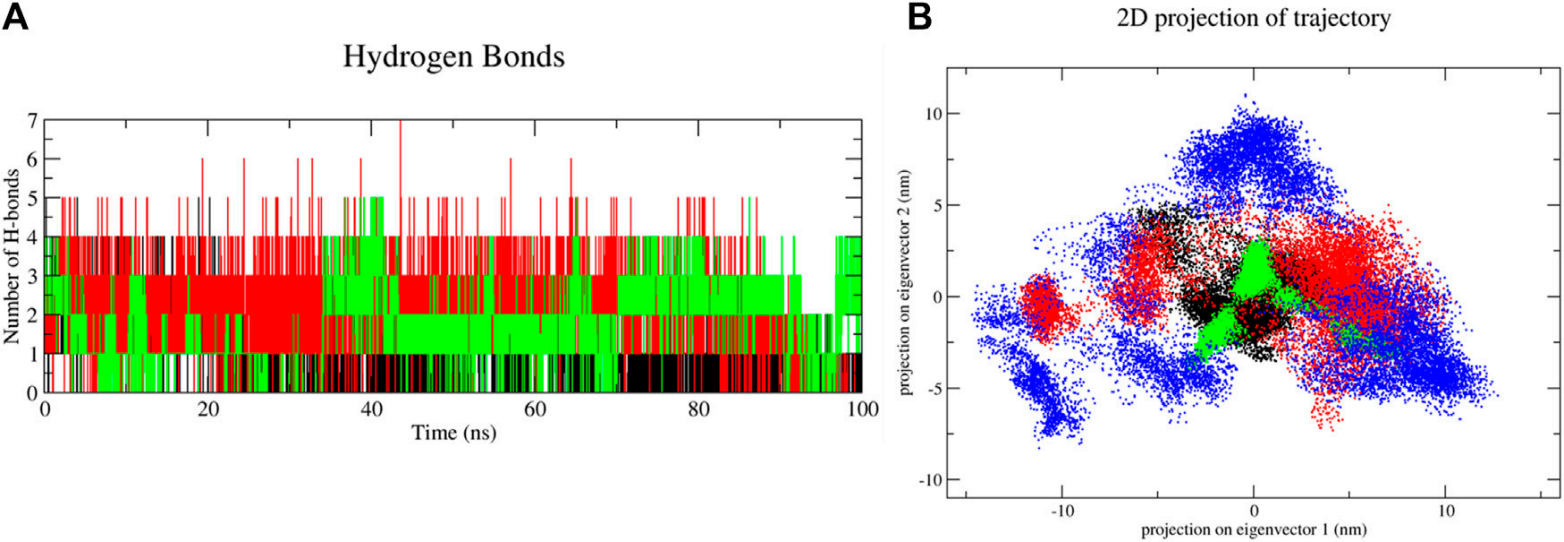
Molecular dynamics simulation results of unbound and first, second and third conformation of digitoxin bound lysozyme complexes, **(A)** Intermolecular hydrogen bonds and **(B)** Essential Dynamic Analysis [Color codes: Lysozyme-black, first conformation of digitoxin bound lysozyme complex-red, second conformation of digitoxin bound lysozyme complex-green and third conformation of digitoxin bound lysozyme complex-blue; For HBond analysis, (Color codes: first conformation of digitoxin bound lysozyme complex-black, second conformation of digitoxin bound lysozyme complex-red and third conformation of digitoxin bound lysozyme complex-green)].

Molecular dynamics simulations have been performed to understand the stability of the complexes described in the molecular docking sections (Das et al., 2018; Sarmah et al., 2022). The results from the molecular dynamics simulations of unbound and three digitoxin bound lysozyme complexes, as reflected in the Root Mean Square Deviation (RMSD), Root Mean Square Fluctuation (RMSF), Radius of Gyration (Rg), Solvent-Accessible Surface Area (SASA), and the Trace of Covariance Matrix (PCA), offer a detailed understanding of the stability, flexibility, compactness, folding properties, dynamic behavior of the lysozyme and its three digitoxin bound complexes. The average RMSD values (Table 3; Figure 9A) for the unbound and three lysozyme-digitoxin complexes offer a snapshot of their structural dynamics during MD simulations. The unbound lysozyme demonstrates a relatively low average RMSD value of 0.137 nm (Table 3), indicative of stable structural maintenance. In contrast, first conformation of digitoxin bound lysozyme exhibits a higher average RMSD value of 0.285 nm, suggesting pronounced conformational changes and increased structural flexibility upon binding. Second conformation of the digitoxin bound lysozyme complex, with an intermediate average RMSD value of 0.180 nm, showcases a moderate level of structural deviation, reflecting a balance between structural stability and structural flexibility. The third conformation of digitoxin bound lysozyme stands out with the highest average RMSD value at 0.403 nm, indicating substantial conformational changes and dynamic rearrangements within this complex. In RMSF analysis (Table 3; Figure 9B), the unbound lysozyme displays an average RMSF value of 0.102 nm, indicating a baseline level of residue flexibility. The first conformation of digitoxin bound lysozyme complex exhibits a slightly higher average RMSF value of 0.119 nm, suggesting localized increases in structural flexibility, potentially attributed to the binding interactions with the ligand molecule. In contrast, the second conformation of the digitoxin bound lysozyme complex displays a lower average RMSF value of 0.088 nm, indicating a more restrained flexibility profile. The third conformation of the digitoxin bound lysozyme complex, with an average RMSF value of 0.124 nm, showcases the highest fluctuation, suggesting pronounced dynamic and conformational changes in specific regions (particularly 70 to 75 residues) of lysozyme. In Rg analysis (Table 3; Figure 9C), the unbound form of lysozyme exhibits an average Rg value of 1.427 nm, suggesting a specific molecular arrangement with a moderate degree of compactness. The first conformation of the digitoxin-bound lysozyme complex, with an average Rg value of 1.460 nm, indicates a slightly increased overall compactness compared to the unbound form of lysozyme structure. The second conformation of the digitoxin-bound lysozyme complex maintains a similar level of compactness, as reflected in its average Rg value of 1.431 nm. In contrast, the third conformation of the digitoxin-bound lysozyme complex stands out with the highest average Rg value at 1.501 nm, suggesting a more extended molecular structure. In SASA analysis (Table 3; Figure 9D), the unbound form of lysozyme showed an average SASA value of 72.377 nm^2^, indicating a certain degree of surface accessibility. The first conformation of the digitoxin-bound lysozyme complex exhibits an increased average SASA value of 77.404 nm^2^, suggesting enhanced exposure of its molecular surface to the solvent environment, potentially as a consequence of binding interactions with the ligand molecule. Conversely, the second conformation of digitoxin bound lysozyme complex displays a slightly decreased average SASA value of 70.653 nm^2^, indicating a reduction in surface accessibility. Notably, the third conformation of digitoxin-bound lysozyme stands out with the highest average SASA value at 78.550 nm^2^, suggesting extended solvent exposure, possibly associated with dynamic structural changes or increased flexibility or pronounced conformational changes. In the context of intermolecular hydrogen bonding analysis (Figure 10A) within digitoxin-bound lysozyme complexes, the observations highlight distinct behavior among different conformational states of lysozyme. Specifically, the first and second conformations of the digitoxin-bound lysozyme complex exhibit a higher number of hydrogen bonds towards the binding site of lysozyme than the third conformation. This finding aligns with the overall trend observed in RMSD, RMSF, Rg, and SASA analyses. The second conformation, displaying increased hydrogen bonding, correlates with lower RMSD value, indicating structural stability, and lower RMSF value, signifying reduced flexibility. The favorable interactions are consistent with a more compact structure, as a lower Rg value suggests. Additionally, the increased SASA suggests better exposure of the binding site. These findings underscore the superiority of the second conformation of the digitoxin-bound lysozyme complex, emphasizing its structural stability, reduced flexibility, and enhanced interactions at the binding site compared to the other conformations. Based on the aforementioned MD simulation analyses, the order of three conformations of digitoxin towards binding site of lysozyme with reference to increase the stability, decrease the flexibility, increase compactness and folding properties is as follows: second conformation of digitoxin > first conformation of digitoxin > third conformation of digitoxin.

**TABLE 3:** Time averaged structural properties obtained from unbound lysozyme and three digitoxin bound lysozyme complexes.

	RMSD (nm)	RMSF(nm)	ROG (nm)	SASA (nm^2^)	Trace of covariance matrix values (nm^2^)
Lysozyme	0.137942	0.102902	1.427236	72.37728	36.1545
Complex_1	0.28554	0.119034	1.460765	77.40411	83.3785
Complex_2	0.180293	0.088691	1.431061	70.65332	30.2327
Complex_3	0.403296	0.124167	1.501492	78.55089	121.145

Complexes 1, 2 and 3 indicates the first, second and third conformation of digitoxin bound lysozyme complexes.

**FIGURE 11:**
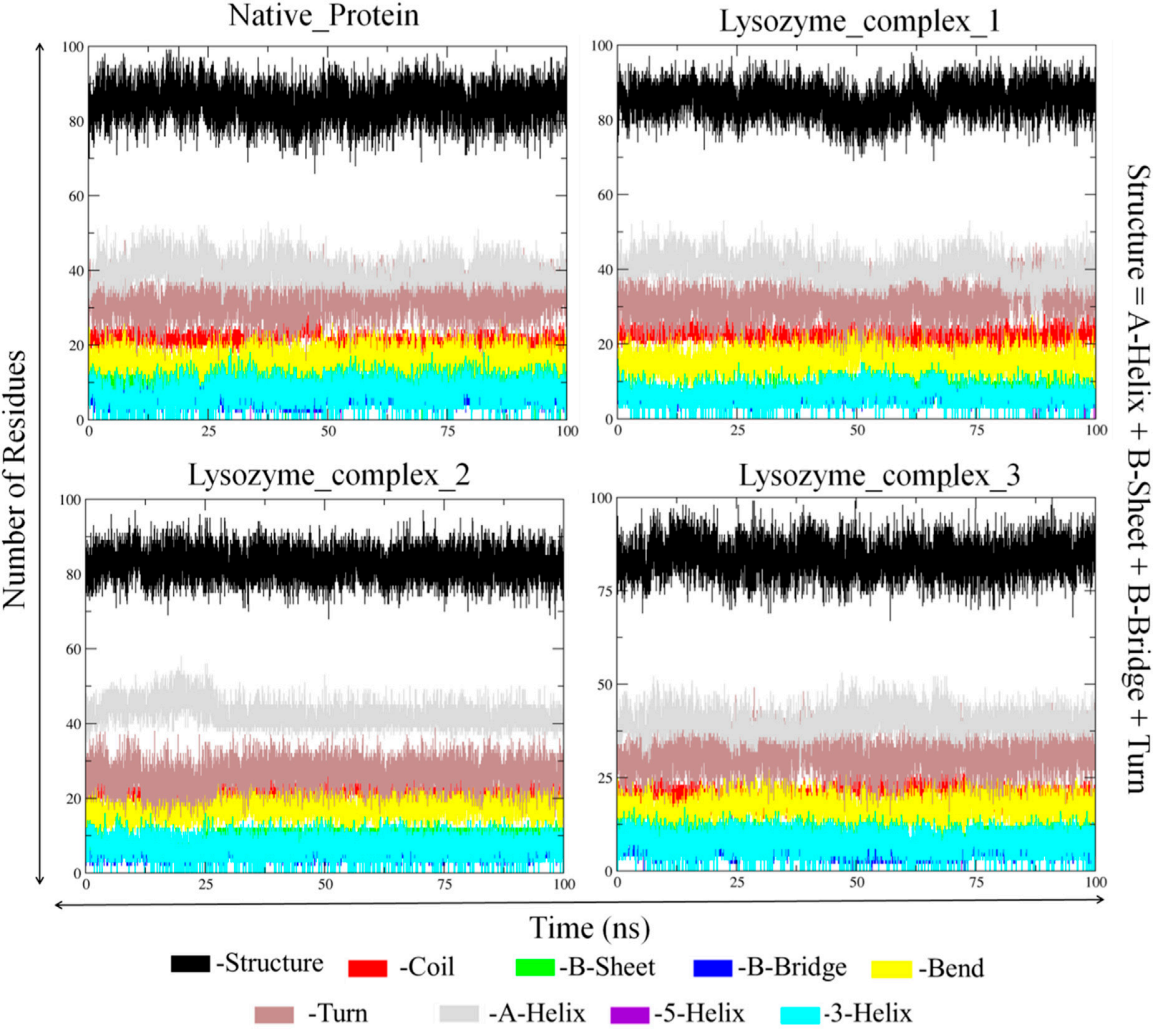
Secondary structural analysis of unbound and first, second and third conformation of digitoxin bound lysozyme complexes.

Apart from the above-mentioned global dynamics analysis, we have additionally performed essential dynamics analysis using Principal Component Analysis (Figure 10B). The PCA is a dimensionality reduction technique which captures essential and biological relevant motions from the pool of global motions. The unbound form of lysozyme exhibits a moderate trace of covariance matrix values of 36.1545 nm2, suggesting inherent flexibility. The first conformation of the digitoxin bound lysozyme complex, with a higher trace of covariance matrix values of 83.3785 nm2, indicates pronounced correlated motions, potentially reflecting dynamic changes upon binding with ligand molecule. The second conformation of the digitoxin bound complex shows a lower trace of covariance matrix values of 30.2327 nm2, suggesting less tightly coupled structural dynamics. Notably, the third conformation of digitoxin-bound lysozyme stands out with the highest trace of covariance matrix values of 121.145 nm2, signifying extensive and strongly correlated motions, underscoring its unique and highly cooperative structural rearrangements during the MD simulations. The secondary structural analysis (Table 4; Figure 11) outlines the conformational changes observed in lysozyme when bound to digitoxin across three distinct conformations (Conformation 1, Conformation 2, and Conformation 3). Lysozyme undergoes subtle alterations in its secondary structural elements upon digitoxin binding. Notably, the coil, beta-sheet, and beta-bridge conformations remain relatively stable. However, slight variations are observed in bend and turn conformations, suggesting a nuanced flexibility in lysozyme’s structure when influenced by digitoxin. The α-helix and 3_10_-helix conformation exhibits minor changes, reflecting the protein’s adaptability. These findings imply that digitoxin induces subtle but discernible shifts in lysozyme’s conformation, potentially influencing its functional dynamics. These minor alterations highlight the specific influence of digitoxin on lysozyme’s secondary structure.

**TABLE 4:** Secondary structural analysis of unbound and three digitoxin bound lysozyme complexes.

Secondary structural elements	Lysozyme	First conformation of digitoxin bound lysozyme complex	Second conformation of digitoxin bound lysozyme complex	Third conformation of digitoxin bound lysozyme complex
**Coil**	0.16	0.17	0.17	0.16
**B-Sheet**	0.06	0.06	0.07	0.06
**B-Bridge**	0.04	0.04	0.04	0.04
**Bend**	0.12	0.12	0.14	0.13
**Turn**	0.24	0.24	0.20	0.24
**α-Helix**	0.31	0.31	0.33	0.31
**3**_**10**_ **-Helix**	0.06	0.05	0.05	0.06

The bold values are secondary structural elements of the protein.

## 4 Conclusion

The importance of digitoxin as a cardiac glycoside and its limitation owing to the side effects associated with its high dose directed us to study its interaction with lysozyme as a potential carrier. There was a strong interaction between lysozyme and digitoxin which increased on increasing the temperature. The interaction was supported by mainly hydrophobic forces with some contrition of hydrogen bonding. Digitoxin has a small impact on the secondary structure of lysozyme by which it partially unfolded the latter. Both experimental and *in silico* investigations were in good agreement with each other and studies show that lysozyme could be used in the drug delivery formulation for developing the suitable carrier for digitoxin.

## Data Availability

The original contributions presented in the study are included in the article/Supplementary material, further inquiries can be directed to the corresponding author.
